# Detailed analysis of deformation potentials with application in orbital-free density functional theory

**DOI:** 10.1107/S2052520621004686

**Published:** 2021-07-14

**Authors:** Kati Finzel

**Affiliations:** a Faculty of Chemistry and Food Chemistry, Technische Universität Dresden, Bergstraße 66c, 01069 Dresden, Germany

**Keywords:** quantum crystallography, orbital-free density functional theory, Pauli potential, bifunctional approach, deformation potentials

## Abstract

A detailed analysis of the recently published deformation potentials for application in orbital-free density functional theory is given. Since orbital-free density functional theory is a purely density-based description of quantum mechanics, it may in the future provide itself useful in quantum crystallography as it establishes a direct link between experiment and theory via a single meaningful quantity: the electron density. In order to establish this goal, sufficiently accurate approximations for the kinetic energy have to be found. The present work is a further step in this direction. The so-called deformation potentials allow the interaction between the atoms to be taken into account through the help of their electron density only. It is shown that the present ansatz provides a systematic pathway beyond the recently introduced atomic fragment approach.

## Abstract 2

This work presents a detailed analysis of the recently published deformation potentials for application in orbital-free density functional theory, that are able to take the interaction between atoms into account through the help of their electron densities only. It is shown that the present ansatz provides a systematic pathway beyond the recently introduced atomic fragment approach.

## 1. Introduction

Quantum crystallography (Massa *et al.*, 1995 ▸, 1999 ▸) is a vividly evolving field at the edge of quantum mechanics and crystallography combining the strengths of each individual field for enhancing the descriptive power of the model (Macchi *et al.*, 2015 ▸; Grabowsky *et al.*, 2017 ▸; Genoni *et al.*, 2018 ▸; Macchi, 2020 ▸). Usually, those strategies rely on sophisticated formalisms [due to subtle but important theoretical aspects (Coleman, 1963 ▸; Schmider *et al.*, 1992 ▸)] matching the experimental data to a density matrix (Gillet & Becker, 2004 ▸) or wavefunction approach (Jayatilaka, 1998 ▸) since many chemical bonding descriptors such as the electron localization function (ELF) (Becke & Edgecombe, 1990 ▸; Savin *et al.*, 1992 ▸) or the electron localizability indicator (ELI) (Kohout, 2004 ▸; Kohout *et al.*, 2004 ▸, 2005 ▸, 2008 ▸) require the first-order density matrix or the pair-density as input. The use of wavefunction approaches allows the insertion of further theoretical concepts such as Extremely Localized Molecular Orbitals (Sironi *et al.*, 2007 ▸), which provides a link to orbital-based interpretation of chemical bonding as well as to further methodological development due to their extremely localized nature and thus, their expected transferability.

However, orbitals are – although admittedly useful – artificial objects solely born from our own conception. There are prominent concepts within the field of quantum chemical topology (Popelier & Aicken, 2003 ▸) providing insight into chemical bonding analysis based on the electron density only. The *quantum theory of atoms in molecules* (QTAIM) (Bader, 1990 ▸) is probably the most well-known representative in the field, but also the source function (Gatti *et al.*, 2003 ▸) is a purely density-based indicator for chemical bonding. Additionally, extensive efforts have been undertaken in order to extract chemical bonding information from experimental densities by employing approximate kinetic energy densities from the gradient expansion (Tsirelson & Stash, 2002*b* ▸,*a* ▸,*c* ▸; Stash & Tsirelson, 2005 ▸; Tsirelson *et al.*, 2013 ▸) or via inhomogeneity measures of the electron density (Wagner & Kohout., 2011 ▸; Finzel *et al.*, 2012 ▸) using ω-restricted space partitioning (Martín Pendás *et al.*, 2012 ▸; Kohout, 2016 ▸). However, care must be taken when employing those indicators as they may markedly differ from their pure quantum mechanic counterparts due to their approximate nature.

The aim of this work is to present a new method, namely the bifunctional approach, providing a purely density-based treatment of quantum mechanics for possible future application in the field of quantum crystallography. Since the method is based on the electron density only, it avoids the detour of the above-mentioned matching formalisms to density matrices or wavefunctions. The new method falls into the field of orbital-free density functional theory (OF-DFT) (Wang & Carter, 2000 ▸; Ho *et al.*, 2008 ▸; Shin & Carter, 2014 ▸; Witt *et al.*, 2018 ▸; Lehtomäki *et al.*, 2014 ▸; Ghosh & Suryanarayana, 2016 ▸). Although founded in 1964 by the famous Hohenberg–Kohn theorems (Hohenberg & Kohn, 1964 ▸), progress in this field was hampered due to the lack of sufficiently accurate kinetic energy functionals (Karasiev & Trickey, 2015 ▸). First attempts were made with gradient expansion techniques, which until now remain the most common research line in the field (Thomas, 1927 ▸; Fermi, 1928 ▸; von Weizsäcker, 1935 ▸; Kirzhnits, 1957 ▸; Hodges, 1973 ▸; Murphy, 1981 ▸; Yang, 1986 ▸; Yang *et al.*, 1986 ▸; Lee & Ghosh, 1986 ▸; Kozlowski & Nalewajski, 1986 ▸; Lee *et al.*, 1991 ▸; Thakkar, 1992 ▸; Liu & Parr, 1997 ▸; Tran & Wesolowski, 2002 ▸; Ayers *et al.*, 2002 ▸; Chai & Weeks, 2004 ▸; Ghiringhelli & Delle Site, 2008 ▸; Lee *et al.*, 2009 ▸; Ghiringhelli *et al.*, 2010 ▸; Salazar *et al.*, 2016 ▸; Ludeña *et al.*, 2018 ▸). As nicely shown by Trickey and co-workers (Trickey *et al.*, 2011 ▸; Karasiev *et al.*, 2014 ▸; Karasiev & Trickey, 2015 ▸) parameterization of generalized-gradient approximations must be performed with care, otherwise these approximations run the risk of producing negative contributions to the Pauli kinetic energy.

The Pauli kinetic energy is a concept that goes back to March (1986 ▸), who defined it as the difference between the full kinetic energy and the von Weizsäcker part (von Weizsäcker, 1935 ▸), which is analytically known and can be seen as the kinetic energy of a bosonic system in its ground-state (having the same density like the fermionic system). Therefore, the Pauli kinetic energy is interpreted as the *extra* kinetic energy necessary to move the electrons into their individual orbitals. As such the Pauli kinetic energy is always of positive nature. Since the Pauli kinetic energy represents the only unknown part of the full kinetic energy, it has been subject of intense theoretical studies (March, 1986 ▸; Levy & Ou-Yang, 1988 ▸; Nagy, 1991 ▸; Nagy & March, 1991 ▸; Nagy & March, 1992 ▸; Holas & March, 1995 ▸; Amovilli & March, 1998 ▸; Nagy, 2008 ▸, 2010 ▸, 2011 ▸; Tsirelson *et al.*, 2013 ▸; Kraisler & Schild, 2020 ▸; Kocák *et al.*, 2020 ▸).

The present work also contributes in this direction. It has been shown recently that reliable approximations for the Pauli kinetic energy can be obtained via bifunctional formalism, involving the electron density and an approximate Pauli potential employing the bare atomic fragment approach (Finzel, 2018*a* ▸,*b* ▸, 2019 ▸, 2020 ▸) and a so-called deformation potential that takes the interaction between two atoms into account (Finzel, 2021 ▸). The present work is a direct follow-up paper of the latter publication (Finzel, 2021 ▸), in which the recently proposed ansatz is subjected to further investigations. Therefore, a detailed analysis of those deformation potentials is given here. It is shown how they work and why they work, and where additional improvements can be expected.

## 2. Theory

In contrast to density functional development in the context of Kohn–Sham density functional theory (KS-DFT), aiming to approximate the electron–electron repulsion, namely the exchange-correlation part, the target in OF-DFT is to approximate the kinetic energy for the system of interest. Following the Hohenberg–Kohn theorems (Hohenberg & Kohn, 1964 ▸), the total electronic energy *E* of a system can be expressed as a functional of the electron density ρ: 

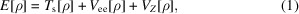

where 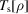 is the non-interacting kinetic energy, *V*_ee_[ρ] is the Coulomb repulsion between the electrons and 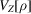 is the electron–nuclear attraction energy. Strictly speaking, equation (1) should refer to the full kinetic energy ( 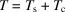 ) consisting of the non-interacting kinetic energy *T*_s_, originating from KS theory, and a correction *T*_c_. The latter term, however, has been shown to be of minor magnitude (Görling & Ernzerhof, 1995 ▸) and is, therefore, usually merged with the exchange-correlation part of the electron–electron interaction. Thus, *T*_s_ is directly introduced in equation (1) as the scaling properties of the non-interacting kinetic energy will be explicitly addressed later.

The electron–nuclear attraction energy 

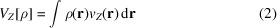

is known exactly as electron density functional by means of the electron density and the nuclear potential of a molecule 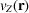 = 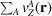 , which is given by the superposition of all atomic nuclear potentials 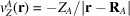 , where 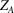 is the nuclear charge and **R**_*A*_ is the nuclear coordinate. In the context of density functional theory (DFT), the electron–electron repulsion *V*_ee_[ρ] is frequently split into the Hartree energy 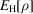 and the exchange-correlation energy 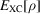 . The Hartree term is usually interpreted as the classical part of the electron–electron repulsion, and it is given by: 

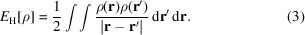

As pointed out at the beginning of this section, in OF-DFT methods the focus is set on approximating the kinetic energy and thus, functional approximations for the exchange-correlation part are generously accepted. Therefore, for simplicity reasons the exchange-correlation part is expressed as local exchange energy 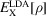 (Hohenberg & Kohn, 1964 ▸) only:

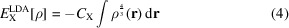

with 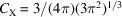 ≈ 0.73856.

As mentioned in the *Introduction* [Sec sec1], the non-interacting kinetic energy 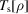 can be regarded as been constructed from a bosonic part, the von Weizsäcker term *T*_W_ (von Weizsäcker, 1935 ▸), and a remainder, the Pauli kinetic energy 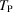 , which consequently, is defined as the difference (March, 1986 ▸):

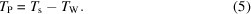

Based on the viewpoint that the von Weizsäcker kinetic energy *T*_W_ is the kinetic energy for a bosonic system in its ground-state (with the actual fermionic density), an analytical expression for the von Weizsäcker kinetic energy density 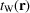 in terms of the electron density can easily be derived: 

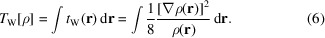

Accepting the approximations for the exchange-correlation energy, the Pauli kinetic energy *T*_P_ is the only unknown functional expression for a purely orbital-free description of quantum mechanics.

As in recently published papers (Finzel, 2018*a* ▸, 2019 ▸, 2020 ▸, 2021 ▸), the Pauli kinetic energy is evaluated from the so-called bifunctional expression: 

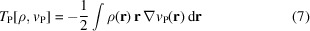

involving both the electron density ρ(**r**) and the Pauli potential 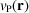 as two separate variables.

A bifunctional expression is obtained by exploiting the homogenous scaling behavior of a functional and further neglecting the density dependence of the respective functional derivative. For a functional *F* which obeys homogeneous scaling behavior: 

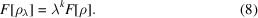

with the homogeneously scaled electron density 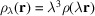 , whereby λ is a parameter and *k* is the respective scaling constant (*k* = 2 in the case of *T*_s_), the functional value can equally be obtained from (Levy & Perdew, 1985 ▸): 

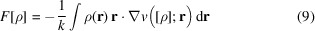

where 

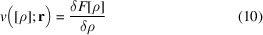

is the functional derivative of *F*. Note that the integral kernel in equation (9) is position dependent, the integral value, however, is not. In the above context, the functional derivative is a true functional derivative 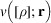 , explicitly given in terms of ρ when the functional expression of *F*[ρ] is given analytically in terms of the electron density. In this case the functional value can, of course, be obtained from the density functional *F*[ρ] alone. The trick in the bifunctional formalism is to suppress the density dependence of the potential, now being a formal functional derivative *v*(**r**). Note that, although an analytical density dependence of the potential can be suggested, for example, as a possible update of the potential with respect to density changes, those analytical dependencies are not exploited in order to obtain the corresponding parent functional. Thus, the formal functional derivative does not have to obey scaling rules, as the functional value from the properly scaling – yet unknown – functional expression can always be obtained from 

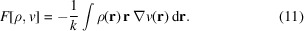

Note that the above equation is a bifunctional expression [in contrast to equation (9)] as it depends on two separate variables ρ and *v*. Thus, based on the homogenous scaling behavior and the corresponding formulas (Levy & Perdew, 1985 ▸) the bifunctional expression allows the extraction of the energy value of the otherwise unknown functional expression. As a consequence, the bifunctional expression provides exactly the KS Pauli kinetic energy, when the molecular electron density and the molecular Pauli potential are inserted into equation (7). The KS Pauli potential of the molecule is only known in terms of the molecular KS eigenfunctions 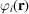 and their respective eigenvalues 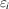 (Levy & Ou-Yang, 1988 ▸): 

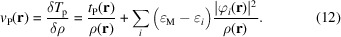

In the above equation, the sum runs over all occupied eigenfunctions and 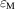 is the highest occupied eigenvalue of the system. The Pauli kinetic energy 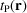 is given by: 

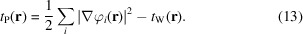

Obviously, in an orbital-free formalism, the KS eigenfunctions and eigenvalues are of course not available. However, as recently shown (Finzel, 2021 ▸), sufficiently accurate approximations for the molecular Pauli potential can be found in order to properly describe chemical bonding by choosing the following ansatz 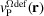 for the Pauli potential: 

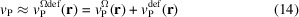

employing the bare atomic fragment approach 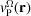 : 

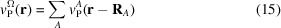

and a so-called deformation potential 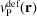 . 

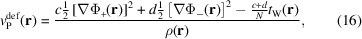

that is based on the constructive 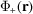 and destructive 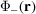 interactions between the atoms. In the above equation *c* and *d* are the number of constructive and destructive electron sharing, respectively, and *N* is the total number of electrons in the system. The respective atomic interactions 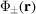 are expressed via the following ansatz: 

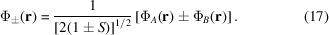

where *S* is the overlap between the functions 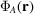 and 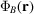 . The individual atomic contributions 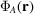 are given by: 

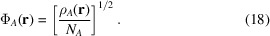

where *N*_*A*_ and 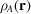 are the number of electrons and the electron density of atom *A*, respectively. Finally, the Pauli kinetic energy is obtained from the bifunctional expression: 

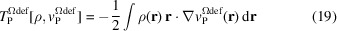

and the total electronic energy is given by 

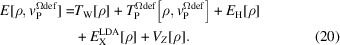




## 3. Computational details

In the current approach, the molecular electron density is given by a simple monopole expansion using atom-centered squared real-type node-less Slater functions. Core regions are described by 1*s*-type functions: 

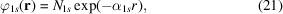

whereas valence regions are modeled by 2s-type functions: 

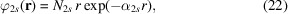

with *N*_1*s*_ and *N*_2*s*_ being the respective normalizations constants. Note that node-less Slater functions are not orbitals, but serve to expand the electron density.

In the present work, energy minimization has been performed by optimizing the respective exponents α_1*s*_ and α_2*s*_, while keeping the corresponding shell occupations fixed [for details see Finzel (2021 ▸)].

KS calculations (molecular data for comparison and closed-shell atoms for the bare atomic fragment approach) were performed with the *ADF* (Software for Chemistry & Materials, 2017 ▸) program at LDA (Xonly) level using the QZ4P basis sets.

## 4. Results and discussion

Equation (14) provides a model for the molecular Pauli potential that explicitly includes the interaction between the atoms by means of the deformation potential, which relies on the number of constructive and destructive electron sharing. Although the underlying idea goes back to molecular orbital (MO) theory, referring to the number of bonding versus anti-bonding orbitals, the proposed ansatz, in principle, allows for any real non-negative number of *c* and *d* (including non-integer values) and can be based on any convenient model that measures electron sharing. In this work, integer numbers that are multiples of two (accounting for doubly occupied molecular orbitals in the sense of MO theory) are tested. As will be shown later, the influence of *c* and *d* on the resultant bond distances is systematic, thereby allowing for valid interpolation between the chosen numbers of *c* and *d*.

The first test case with *c* = 0 and *d* = 0 is the bare atomic fragment approach itself, in which case the equilibrium bond distances directly follow the size of the core shells, which decrease from Li to Ne and thus, yield a very short bond length in the case of Ne_2_ [for a detailed explanation see Finzel (2021 ▸)]. The construction of a deformation potential with *c* = 2 and *d* = 0 means that one electron pair interacts constructively, while there are no destructive terms at all. This model can, of course, be tested for all dimers in order to show the systematic behavior of the proposed model, but only in the case of Li_2_ is this model in accordance with the MO concept (Li–Li having one single shared electron pair). Increasing *c* while keeping *d* equal to zero signifies an increasing constructive electron sharing with one, two, three, four and five electron pairs for *c* = 0, 2, 4, 6, 8 and 10, respectively. The latter are of course not realized within the second-row homonuclear dimers, but with respect to the methodology itself it is worth investigating in order to test whether this will lead to a systematic decrease in the bond lengths. Deformation models with *d* = 2 share one electron pair of destructive nature. In the sense of MO theory, *c* = 2 and *d* = 2 would be the electronic graph describing Be_2_ with one constructive and one destructive electron pair. According to MO theory the following next dimers B_2_, C_2_ and N_2_ would be characterized by *d* = 2 (in all cases) and *c* = 4, 6 and 8, respectively, but of course other combinations of *c* and *d* are valid for testing. Following the MO concept beginning from O_2_ additional destructive terms have to be added. Thus, *c* = 8 for O_2_, F_2_ and Ne_2_, and *d* = 4, 6 and 8, respectively, for the design of MO-compatible deformation potentials. However, as stated before all combinations of *c* and *d* are tested in order to investigate whether or not the proposed ansatz behaves systematically (which is of significant importance for the applicability of further possible models for *c* and *d*).

**Figure 1:**
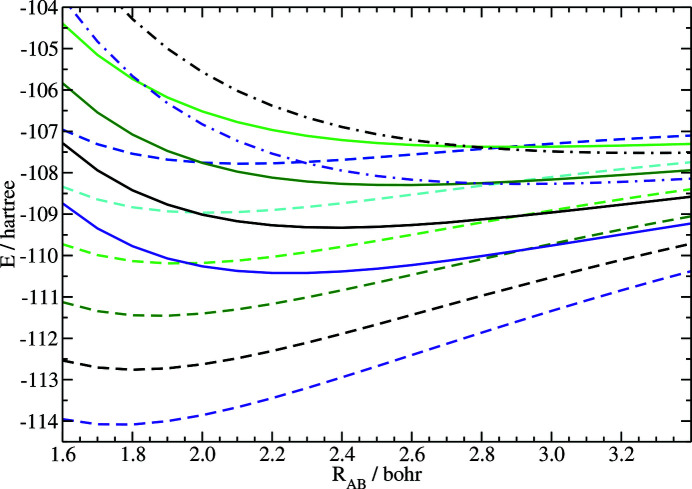
Total energy of N_2_ as a function of the internuclear distance from OF-DFT using various deformation potentials. Dashed lines: *d* = 0; full lines: *d* = 2; dashed–dotted lines: *d* = 4. Dark blue: *c* = 0; light blue: *c* = 2; light green: *c* = 4; dark green: *c* = 6; black: *c* = 8; violet: *c* = 10.

In order to investigate the reliability of the proposed ansatz, deformation potentials for various combinations of constructive and destructive interaction terms were generated and their performance with respect to the chemical bonding was tested. For example, those bonding curves, showing the total energy as a function of the internuclear distance, are depicted for the N_2_ molecule in Fig. 1 ▸. As can be seen from the figure, the proposed ansatz yields reasonable bonding curves over a large set of possible input for *c* and *d*.

That aspect is noteworthy, since the design of kinetic energy density functionals providing reasonable energy differences, *e.g.* for a molecule with varying internuclear distance, is extremely challenging, while so-called single-shot functionals (aiming to represent the kinetic energy for a special system of interest, *e.g.* at the equilibrium bond distance) can conveniently be obtained numerically by inversion of KS equations or with the help of an appropriate parameterization. In contrast, the proposed ansatz does not require *ad hoc* parameterization. However, specific chemical knowledge about the number of valence electrons of the participating atoms is needed in order to determine meaningful choices for *c* and *d*. In return, meaningful choices of *c* and *d* yield bound systems. Additionally, bond lengths obtained from those deformation potentials all lie within a reasonable range, *e.g.* they vary from 1.8 bohr to 3.3 bohr in the case of the N_2_ molecule. Notably, changes in the equilibrium bond length are remarkably systematic. Increasing the number of constructive terms (for a fixed number of *d*) (see for example energy curves depicted by dashed lines) yields decreasing bond distances [follow the data shown in blue (*c* = 0) to the data shown in violet (*c* = 10) (Fig. 1 ▸)], while increasing the number of destructive terms (for fixed *c*) yields increasing bond distances [see for example data represented in black (*c* = 8) with increasing number of destructive terms, *d* = 0 depicted by dashed lines, *d* = 2 depicted by full lines, and *d* = 4 depicted by dashed-dotted lines (Fig. 1 ▸)]. This is a favorable outcome with respect to chemical bonding theory. Adding more constructive terms favors chemical bonding, while adding destructive terms correspondingly weakens the bond. The proposed model, thus, provides systematic and predictable results for a given choice of deformation potential. Those aspects are, on the one hand, given by the systematic construction of the deformation potentials and, on the other hand, the impact of such potentials on its ability to describe chemical bonding can equally be rationalized by visualization.

**Figure 2:**
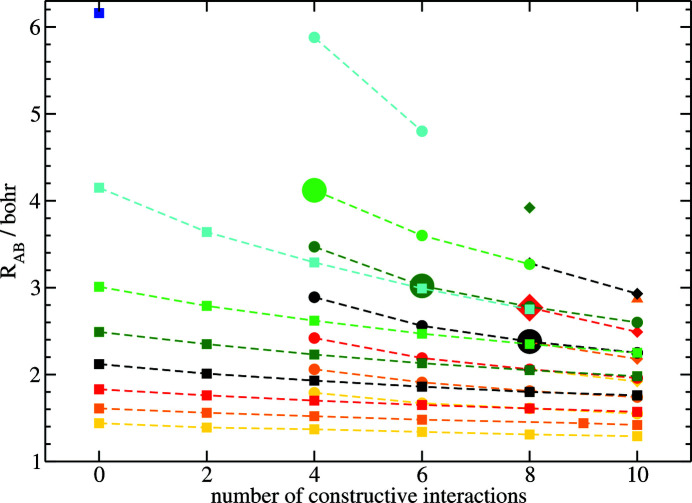
Equilibrium bond length for second-row homonuclear dimers from OF-DFT using various deformation potentials as a function of constructive interaction terms. Squares: no destructive terms, circles: two destructive terms, diamonds: four destructive terms, triangles: six destructive terms. Yellow: Ne_2_; orange: F_2_; red: O_2_; black: N_2_; dark green: C_2_; light green: B_2_; light blue: Be_2_; dark blue: Li_2_. Equilibrium bond lengths from deformation potentials in accordance with MO concept are highlighted by large symbols, hereby B_2_ *R_AB_* = 4.12 bohr (shown by light-green circle), C_2_ *R_AB_* = 3.02 bohr (shown by dark-green circle), N_2_ *R_AB_* = 2.38 bohr (shown by black circle), and O_2_ *R_AB_* = 2.77 bohr (shown by red diamond). The equilibrium bond length for N_2_ has recently been reported by Finzel (2021 ▸).

Fig. 2 ▸ presents the equilibrium bond distances for the second-row homonuclear dimers as a function of the number of constructive terms. As can be seen from the figure, the observations made for N_2_ are valid for all examined test cases, meaning that with increasing *c* the respective bond distance decreases accordingly. Additionally, note also that the bond-length contraction behaves in a systematic way. For bond distances with *d* = 0 the contraction becomes more pronounced with decreasing nuclear charge, see for example the bond lengths depicted with square icons beginning from Ne_2_, shown in yellow, to Be_2_, shown in light blue (Fig. 2 ▸). Larger atoms, thus, exhibit a more compressible behavior in the proposed model. Moreover, for one and the same dimer *X*_2_, the bond-length contraction for increasing *d* is more pronounced, which is also due to the size effect. The respective energy minima with higher values for *d* are shifted towards larger bond distances and the impact of increasing numbers for *c* is higher in those regions as the energy curve is much flatter here.

Thus, due to their systematic nature the recently introduced deformation potentials enable substantial improvement of the description of chemical bonding within the second-row homonuclear dimers compared to the bare atomic fragment approach. As can be seen from Fig. 2 ▸, the bare atomic fragment approach yields systematically decreasing bond lengths for *X*_2_ when going from Li (shown in dark blue) to Ne (shown in yellow), see entries at the very left side of the figure where *c* = 0 and *d* = 0 (data represented by squares). In the bare atomic fragment approach, only the effect due to the Pauli repulsion of a given atomic density with the core electrons of its neighboring atoms is taken into account, not the nature of the interaction of the valence densities. Since the core regions decrease in size from Li to Ne, the respective Pauli repulsion decreases, and consequently, the resultant bond distance decreases. As a matter of fact, adding constructive terms to the bare atomic fragment approach yields an even smaller bond length, and thus, Ne_2_ with *d* = 0 exhibits the shortest bond distances for the examined test cases (see the data depicted by yellow squares in Fig. 2 ▸). As shown by the data, a systematic lengthening of bond distances is obtained by adding more destructive terms. Different values of *d* are represented by different symbols, *d* = 0 depicted by squares, *d* = 2 depicted by circles and *d* = 4 depicted by diamonds. Usually deformation potentials with *d* = 6 or higher yield unbound atoms. Therefore, F_2_ and Ne_2_ are unbound when *c* and *d* are chosen according to MO theory [for further details see Finzel (2021 ▸)].

In summary, the calculated equilibrium bond lengths build a consistent set, which is in accordance with our expectation from traditional electronic structure theory (Kutzelnigg, 2002 ▸). Based on the knowledge of molecular orbital (MO) theory, prominent experimental findings like the bond-length contraction for N_2_ within the list of the second-row homonuclear dimers can be rationalized. Recall that the density is represented by a simple monopole expansion in this work, and thus, the experimentally observed bond-length contraction is reproduced without the necessity of introducing angular quantum numbers (*s*-, *p*-, *d*-orbitals) for the participating atoms. In Fig. 2 ▸, deformation potentials with *c* and *d* in accordance with MO theory are labeled by large icons, hereby B_2_ *R_AB_* = 4.12 bohr (shown by a light-green circle), C_2_ *R_AB_* = 3.02 bohr (shown by a dark-green circle), N_2_ *R_AB_* = 2.38 bohr (shown by a black circle), and O_2_ *R_AB_* = 2.77 bohr (shown by a red diamond) (calculations for Li_2_ do not converge, and Be_2_, F_2_ and Ne_2_ are unbound within that model). However, the data nicely reveals the bond-length contraction from B_2_ (shown in light green) via C_2_ (shown in dark green) to N_2_ (shown in black), which is due to subsequently increasing constructive interaction, together with the lengthening of the internuclear distance from N_2_ to O_2_ (data for O_2_ is shown in red), since the number of destructive terms *d* increases from *d* = 2 in N_2_ to *d* = 4 in O_2_. Thus, the electron counting rules (from the MO concept) in connection with spherical atoms are sufficient to explain the experimentally observed bond-length contraction.

**Figure 3:**
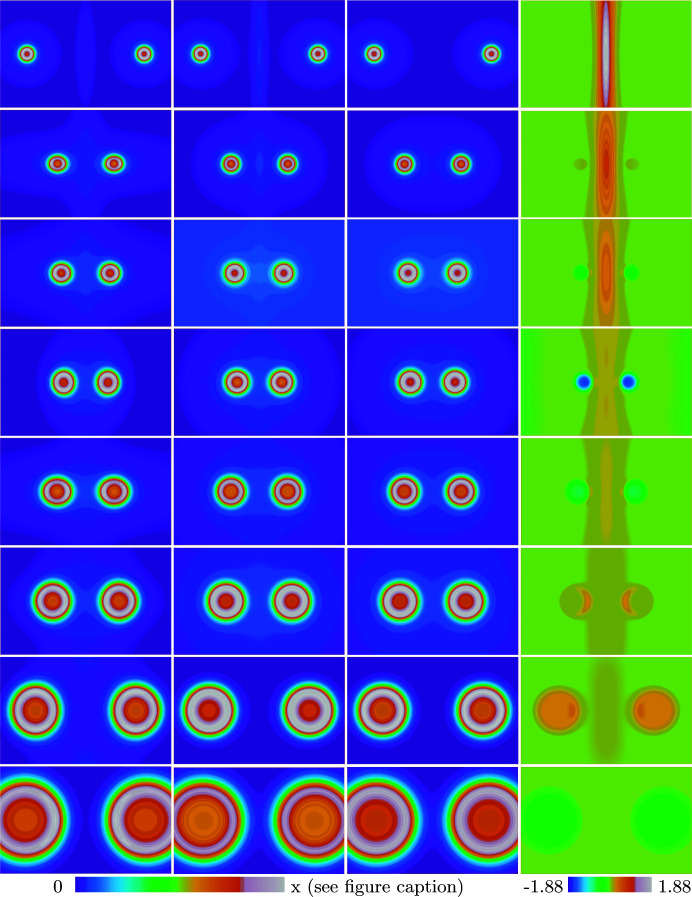
Pauli potential (PP) and components for second-row dimers. The first column depicts the molecular PP evaluated from Kohn–Sham orbitals (Finzel, 2021 ▸) whereas the second column depicts the orbital-free approximation 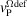 together with its components 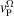 (Finzel, 2021 ▸) and 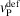 shown in columns three and four, respectively. First row: Ne_2_ color scale from 0.0 (blue) to 41.0 (white). Second row: F_2_ color scale from 0.0 (blue) to 36.0 (white). Third row: O_2_ color scale from 0.0 (blue) to 26.0 (white). Fourth row: N_2_ color scale from 0.0 (blue) to 18.5 (white). Fifth row: C_2_ color scale from 0.0 (blue) to 14.3 (white). Sixth row: B_2_ color scale from 0.0 (blue) to 8.9 (white). Seventh row: Be_2_ color scale from 0.0 (blue) to 5.2 (white). Eighth row: Li_2_ color scale from 0.0 (blue) to 2.4 (white). Orthoslices are shown within the range of 5×8 bohr for all dimers.

As shown in recently published work (Finzel, 2021 ▸), the currently proposed model of deformation potentials slightly overestimates the destructive interaction terms, consequently yielding bond lengths that are somewhat too long compared to the corresponding experimental data. This aspect can also be visually observed by comparison of the approximate molecular Pauli potentials 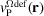 and the orbital-based KS Pauli potentials (PP) at the equilibrium bond distances obtained from KS/LDA/QZ4P calculations. The data are compiled in Fig. 3 ▸, together with the components 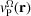 and 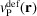 , shown in columns three and four, respectively. At first glance, the close similarity between the orbital-based KS PP, shown in the first column, and the approximate molecular PP 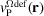 , shown in the second column, can be noticed. Apparently, the recently proposed ansatz is able to mimic the molecular KS PP not only in the core regions, but also in the bonding regions, where minor characteristics are of high importance. However, by careful visual inspection a slight imbalance can be noticed. As can be seen, the impact of the destructive terms is somewhat over-charged in the current model. Values of the approximate PP at the bond critical point are slightly higher compared to their KS data. Consequently, in this region the gradient of the approximate PP is higher than the corresponding gradient for the KS PP, and the Pauli repulsion is more pronounced. This effect is particularly noticeable in Ne_2_ and F_2_, but the general trend applies to all second-row homonuclear dimers. The data in Fig. 3 ▸, thus, reveals that by careful inspection of approximate deformation potentials and subsequent engineering, systematic improvements can be obtained.

A proof-of-concept is given by analyzing columns three and four in Fig. 3 depicting the bare atomic fragment approach and the deformation potential for the second-row homonuclear dimers *X*_2_ with *X* = Ne, shown in the first row, until *X* = Li, shown in the last row. The reader will note the strong impact of the deformation potential within the core regions in the case of N_2_, shown in the fourth row, and somewhat smaller in the cases of C_2_ and O_2_, depicted below and above, respectively. Although, aimed to approximate the first term 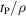 of the orbital-based KS PP, a quantity that is always positive, the approximate deformation potential exhibits negative values within the core regions. This is due to the fact that the approximate deformation potential is not built from the respective eigenfunctions, while the KS Pauli kinetic energy is given in terms of such orbitals. Nevertheless, the full approximate PP 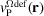 is positive everywhere, a mandatory requirement for appropriate approximations of the Pauli potential (Karasiev & Trickey, 2015 ▸). However, the fact that the deformation potential in the cases of O_2_, N_2_ and C_2_ has a non-negligible contribution in the core regions signifies (already by visual inspection) that in contrast to the bare atomic fragment approach (Finzel, 2019 ▸) (depicted in column three) optimization of the core regions will have an impact on the corresponding OF-DFT calculations and thus, influences the resultant bonding curve.

The above-mentioned aspect has been verified by comparing the chemical bonding curves from valence optimized (optimization of 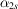 only) and the fully optimized electron-density (optimization of 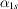 and 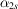 ) calculations. The corresponding equilibrium bond lengths together with the respective dissociation energies are compiled in Table 1 ▸. As can be seen from the data, the bond distances are indeed influenced by the optimization of the core electron density. In all cases the additional optimization leads to smaller equilibrium bond lengths. As can be expected from the data in Fig. 3 ▸, this effect is most pronounced in the case of the N_2_ molecule, where the most prominent bond-length contraction in connection with the core-density optimization is found. The respective dissociation energies behave accordingly, and despite being somewhat high are in good agreement with the concept of multiple bonding, showing that N_2_ has the highest dissociation energy in this model.

## 5. Conclusion

In this work, the recently published deformation potentials with application in orbital-free density functional theory were subjected to a detailed analysis.

In principle, orbital-free density functional theory (OF-DFT) provides a direct link between experimental measurements and quantum theory based on a single quantity with interpretative meaning: the electron density. As such OF-DFT avoids detours via wavefunctions or density matrices. However, in the past there has been no general formalism of OF-DFT in reasonable accordance with orbital-based quantum theory. The present work aims to overcome such shortcomings. It was shown that based on the recently introduced bifunctional approach, sufficiently accurate approximations can be found that allow a reliable description of chemical bonding.

Here, a detailed analysis of those approximations was given. The recently introduced deformation potentials together with their underlying reasonings were presented in detail. It was shown how they work and why they work, and that based on careful inspection, the performance of a given deformation potential can be predicted in advance. Those findings offer a new strategy for systematic improvements in OF-DFT.

## Figures and Tables

**Table 1:** Equilibrium bond length *R_AB_* (in bohr) and dissociation energies *D*_0_ (in hartree) for O_2_, N_2_ and C_2_ from OF-DFT with optimized valence regions (val opt) as well as core and valence optimized electron density (full opt) Valence optimized equilibrium bond length have recently been reported by Finzel (2021 ▸).

	val opt		full opt	
	*R_AB_*	*D*_0_	*R_AB_*	*D*_0_
O_2_	2.77	0.511	2.73	0.663
N_2_	2.38	2.330	2.33	2.631
C_2_	3.02	0.724	2.99	0.809
